# Integrating humanities curricula in medical education: a needs assessment

**DOI:** 10.15694/mep.2017.000090.2

**Published:** 2017-06-05

**Authors:** Anna Taylor, Susan Lehmann, Margaret Chisolm

**Affiliations:** 1 University of Bristol; 2 Johns Hopkins University

**Keywords:** art, humanities, curriculum

## Abstract

This article was migrated. The article was not marked as recommended.

Background: Medicine’s increasing technologic complexities can constrain medical learners’ development of patient-centered communication skills, which can adversely impact patient outcomes. Although humanities-based clinical education interventions encourage reflective practice and promote the practice of holistic patient care, it remains unclear which educational interventions are the most effective.

Methods: A search was conducted in PubMed, utilising the terms ‘humanities’, ‘humanism’, ‘art’, ‘medicine’, ‘narrative medicine’, and ‘medical education’ to identify relevant English-language articles. Discussion with experts yielded further titles, such that 156 articles were reviewed and summarised, with particular focus on those describing novel curricular interventions.

Results: 108/156 (69%) of the articles were commentaries or reflective papers; 48/156 (31%) reported on curricular interventions. Of the latter, the majority incorporated literature or ethics, typically delivered in small-group format. Only ten interventions included impact assessment measures beyond learner satisfaction. Five of these used qualitative evaluations, three used quantitative scales, and two used both.

Discussion: Humanities-based curricular interventions with a focus on literature or ethics were more common than those involving the visual or performing arts. Among the studies that evaluated these curricular interventions, the majority employed qualitative measures. Collaborative teaching between clinicians, arts educators and patients may be considered in order to bridge the gap between science and humanities.

## Background

Learning to take a holistic approach to patient care is more important for medical students today than ever, as an explosion of biomedical discoveries in genetics and pathophysiology are continuously being integrated into medical education ( Pedersen 2010). The loss of empathy among medical students and junior doctors as they progress through training is also a well-described phenomenon ( Pedersen 2010, Stratta 2016), which may negatively impact the therapeutic doctor/patient relationship and effective patient care ( Banerjee 2012, McCullough 2015). There is clearly a need to help trainees retain empathy in order to become more humanistic clinicians ( Batt-Rawden 2013), and educational interventions such as narrative writing can be effective in promoting reflection ( Levine 2008). Other humanities-based curricula that have been shown to enhance reflection involve visual arts, literature and theatre ( Schwartz 2009). However, little is known about the impact and outcome of humanities-based educational interventions in the medical school curriculum.

This paper aims to review the literature regarding the integration of humanities curricula into medical education, including methods of measuring the effectiveness of such interventions. The paper will summarise the key learning points from the literature on this topic and identify any gaps in the literature.

## Methods

A systematic search was conducted in PubMed in September 2015, utilising the terms ‘humanities’, ‘humanism’, ‘art’, ‘medicine’, ‘narrative medicine’, and ‘medical education’ to identify a body of published English-language articles of relevance to the topic of interest. No publication date limits were set. Each article was reviewed and summarised, with particular focus on descriptions of curricula and outcome measures used to evaluate impact.

## Results

The search yielded 163 titles. Discussion with experts in the field yielded an additional 10 articles for review. Of these 173 articles, 17 were excluded because a full text was not available, yielding a total of 156 articles. See Figure 1 for a summary of the results.

Of the 156 articles reviewed, most (108; 69.2%) were commentaries and reflections on the humanities as they relate to medicine. Forty-eight articles described a humanities-based intervention. Twenty-six of these 48 articles did not report any formalised evaluation outcomes ( Goodwin 2015, Kemp 2014, Ortega 2011, Wald 2010, Joachim 2008, Kumagai 2008, Boudreau 2007, Meites 2003, Louis-Courvoisier 2003, Frich 2003, Acuna 2003, Murray 2003, Jones 2003, Hawkins 2003, Wear 2003, Fried 2003, Spike 2003, Krackov 2003, Sirridge 2003, Andre 2003, Montgomery 2003, Kirklin 2003, Rizzolo 2002, Sklar 2002, Downie 1997, Self 1990). Of the 22 articles reporting evaluation outcomes, 12 described learner satisfaction outcomes (del Pozo 2005, Wald 2015, Gurtoo 2013, Abdel-Halim 2012, George 2011, Karnad 1999, Shapiro 2003, Newell 2003, Anderson 2003, Lypson 2002, Bertman 1985, Wilson 1980). Only ten articles included any formal method of evaluation or assessment of impact beyond learner satisfaction. Of those ten articles, five used qualitative techniques to evaluate learners ( Thompson 2015, Ramani 2013, Gulpinar 2009, Wachtler 2006, Bonebakker 2003), three used quantitative measures ( Rodriguez 2013, Wiecha 2008, Wiecha 2002), and two used a combination ( Shapiro 2004, Shapiro 2005). Quantitative measures were typically Likert scales or validated empathy scales (Empathy Construct Rating Scale and the Balanced Emotional Empathy Scale ( Shapiro 2004).

A number of areas within the humanities were utilized in curricula, but the most common type of intervention was one based around poetry, prose or ethics, with fewer interventions using the visual arts or performing arts. Several methods of integrating humanities interventions were described. A common format was small group teaching, and interventions were often facilitated by a professional with humanities experience ( George 2011, Wald 2010, Gulpinar 2009, Boudreau 2007, Wachtler 2006, Karnad 1999, Shapiro 2004, Hawkins 2003, Andre 2003, Shapiro 2003, Newell 2003, Anderson 2003). Only three interventions utilized newer methods of medical education such as websites to curate content, or social media to enable more frequent communication between students ( Wiecha 2002, Wiecha 2008, George 2011).

Two of the educational interventions incorporated humanities teaching into an anatomy course to enable students to begin to understand the patient perspective of illness along with learning techniques of dissection ( Bertman 1985, Rizzolo 2002). Of note, few of the 48 articles described an intervention delivered in a clinical environment or in the presence of a patient, using related humanities material (such as examples from literature describing a patient’s experience of a certain illness) to help students comprehend the impact of different illnesses on patients ( Ramani 2013, Kumagai 2008, Wilson 1980, Gurtoo 2013). Curricula were predominantly delivered in either a didactic or seminar-based format with no patient involvement. One intervention used social media (including Twitter, YouTube and Skype) to augment classroom teaching ( George 2011), while another hosted the content of a humanities clerkship on a website ( Wiecha 2008), enabling students to access materials at times of their choosing.

## Discussion

Assessing the landscape of the literature on the integration of humanities curricula into medical education revealed that the majority of articles were not reports of original research; rather they were opinion pieces discussing the relationship between humanities and medicine, or arguing for the inclusion of humanities teaching within medical education. Few articles described a curricular intervention and, of those that did, a very small minority also included any outcomes measurements on trainee knowledge, attitudes or behaviours. None of the articles assessed the impact of humanities curricula on patients, or evaluated patient care outcomes.

Among the articles that described means of evaluating the effectiveness or impact of humanities interventions, qualitative feedback derived from learner interviews or written feedback was the most commonly used method. Although qualitative methodology enables participants to express themselves more freely and flexibly about issues and experiences that are important to them, it can be difficult to compare open-ended feedback from learners about educational interventions or to determine whether such interventions would be suitable for different population groups. The articles reporting use of a quantitative scale to evaluate impact typically used graded Likert ratings, although one study used the Empathy Construct Rating Scale (ECRS) and the Balanced Emotional Empathy Scale (BEES) ( Shapiro 2004). Although all of these scales use self-reported measures and therefore are subject to bias, they may be considered more objective than open-ended or written feedback, which is vulnerable to variability in interpretation among raters.

Certain challenges were frequently identified throughout the literature. Lack of funding was a commonly cited problem, resulting in humanities curricula that could not be guaranteed a long-term place in medical training. Another common problem was difficulty scheduling the teaching amongst the multitude of other academic commitments held by learners.

This review identified a number of significant gaps in the literature, the most important being a lack of outcome data. This significantly limits the evidence base for the use of humanities in medical training, and which may make it hard to argue for more widespread inclusions in medical education. Future studies should focus on evaluating the impact of humanities-based didactics on trainees, either in the form of qualitative data or using scales already described in the existing literature.

Additionally, many of the educational interventions were undertaken as elective courses, potentially creating a self-selected group of interested learners. This could skew any evaluation results towards a positive impact. Evaluation of humanities curricular interventions that are integrated into the general medical curriculum will be especially valuable in determining impact on the training of physicians. Since the goal of including humanities curricula is to help trainees become more humanistic clinicians, it is important that future studies pursue assessment of whether such a curriculum improves humanistic practice by doctors.

Most of the curricula were described as running separately to biomedical teaching on areas such as pathology, biochemistry or physiology. They were often taught by arts educators, without clinician involvement. This could limit the potential for students to understand how humanities can contribute to all areas of medicine as opposed to simply communication or writing skills. Integrated collaborative teaching, delivered by arts educators together with clinicians and involving an understanding of both the biomedical underpinnings of illness and the experience of illness itself, could help to bridge the gap between science education and humanities education. It could therefore help to illuminate its relevance for facilitating a more holistic understanding of patients.

Finally, although many of the interventions were delivered during medical training, and students therefore participated in separate humanities courses contemporaneously with meeting patients and involving themselves in the ward environment, very few of the interventions actually involved the patients themselves. Including patients in medical education has been shown to enhance the student learning experience ( Jha 2009, Ramani 2013). Speaking to patients, either in the classroom or at the bedside, could help students connect the ‘standard patient’ with classic symptoms described in medical textbooks to the patient read about in assigned literature or viewed in art. Effective humanities teaching also includes time for reflection and focused mentoring to ensure positive learning experiences are gained ( Stern 2008). The human connection engendered in humanities interventions may help preserve empathy felt towards patients, which has been identified previously as an area of importance ( Pedersen 2010, Stratta 2016).

## Conclusion

The role of the humanities within medical education has been extensively discussed in theory, but very little has been done to evaluate its use in practice. This review identified a number of significant gaps in the literature, the most important being a lack of rigorous evaluation of curricular interventions that include outcome measures. Future studies should focus on gaining qualitative and quantitative data regarding impact of curricular interventions on learners and/or patients.

## Take Home Messages

• There is a need to help medical students and junior doctors retain empathy as they progress through training
• Humanities curricula have been shown to enhance reflection
• There is a lack of outcome data demonstrating impact on learner behaviour or the patient experience
• Collaborative teaching between clinicians, arts educators and patients may be considered in order to bridge the gap between science and humanities
• Challenges to long-term integration of humanities curricula include lack of funding and difficulty scheduling
• Future work should include assessment of the outcome of humanities-based educational interventions on medical learner and patient outcomes

## Notes On Contributors

*Anna K. Taylor* is a final year medical student at the University of Bristol. She has five years’ experience in medical education and curriculum development, focusing on global health, diversity training, and a holistic approach to patient care.

*Susan Lehmann* is associate professor of Psychiatry and Behavioral Sciences and Psychiatry Clerkship Director at the Johns Hopkins University School of Medicine in Baltimore, Maryland.

*Margaret S. Chisolm* is associate professor of and Vice Chair for Education in Psychiatry and Behavioral Sciences at the Johns Hopkins University School of Medicine in Baltimore, Maryland.
