# Integrating humanities curricula in medical education: a literature review

**DOI:** 10.15694/mep.2017.000090.2

**Published:** 2018-08-10

**Authors:** Anna Taylor, Susan Lehmann, Margaret Chisolm

**Affiliations:** 1 University of Bristol; 2 University of Manchester; 3 Johns Hopkins University; 4 Johns Hopkins University School of Medicine

**Keywords:** Art, Humanities, Communication, Reflection, Narrative medicine, Medical education, Curriculum, Humanism and professionalism

## Abstract

This article was migrated. The article was marked as recommended.

**Introduction:** Medicine’s increasing technologic complexities can constrain medical learners’ development of patient-centered communication skills, and adversely impact patient outcomes. Although humanities-based clinical education interventions encourage reflective practice and promote the practice of holistic patient care, it remains unclear which educational interventions are the most effective.

**Methods** **:** A search was conducted in PubMed, utilising the terms ‘humanities’, ‘humanism’, ‘art’, ‘medicine’, ‘narrative medicine’, and ‘medical education’ to identify relevant English-language articles. Discussion with experts yielded further titles, such that 156 articles were reviewed and summarised, with particular focus on those describing novel curricular interventions.

**Results:** 108/156 (69%) of the articles were commentaries or reflections; 48/156 (31%) reported on curricular interventions. Of the latter, the majority incorporated literature or ethics, typically delivered in small-group format. Only ten interventions included impact assessment measures beyond learner satisfaction. Five of these used qualitative evaluations; three, quantitative scales; and two, both.

**Discussion:** Humanities-based curricular interventions with a focus on literature or ethics were more common than those involving the visual or performing arts. Among the studies that evaluated these curricular interventions, the majority employed qualitative measures. Collaborative teaching between clinicians, arts educators and patients may be considered in order to bridge the gap between science and humanities.

## Introduction

Learning to take a holistic approach to patient care is more important for medical students today than ever, as an explosion of biomedical discoveries in genetics and pathophysiology are continuously being integrated into medical education ( Pedersen, 2010). The loss of empathy among medical students and junior doctors as they progress through training is also a well-described phenomenon ( Pedersen, 2010; Stratta, Riding and Baker, 2016), which may negatively impact the therapeutic doctor/patient relationship and effective patient care ( Banerjee and Sanyal, 2012; McCullough *et al.*, 2015). There is clearly a need to help trainees retain empathy in order to become more humanistic clinicians ( Batt-Rawden *et al.*, 2013), and educational interventions such as narrative writing can be effective in promoting reflection ( Levine, Kern and Wright, 2008). Other humanities-based curricula that have been shown to enhance reflection involve visual arts, literature and theatre ( Schwartz *et al.*, 2009). However, little is known about the impact and outcome of humanities-based educational interventions in the medical school curriculum.

This paper aims to review the literature regarding the integration of humanities curricula into medical education, including methods of measuring the effectiveness of such interventions. The paper will summarise the key learning points from the literature on this topic and identify any gaps in the literature.

## Methods

A search was conducted in PubMed in September 2015, utilising the terms ‘humanities’, ‘humanism’, ‘art’, ‘medicine’, ‘narrative medicine’, and ‘medical education’ to identify a body of published English-language articles of relevance to the topic of interest. No publication date limits were set. Each article was reviewed and summarised, with particular focus on descriptions of curricula and outcome measures used to evaluate impact.

## Results

**Figure 1.**
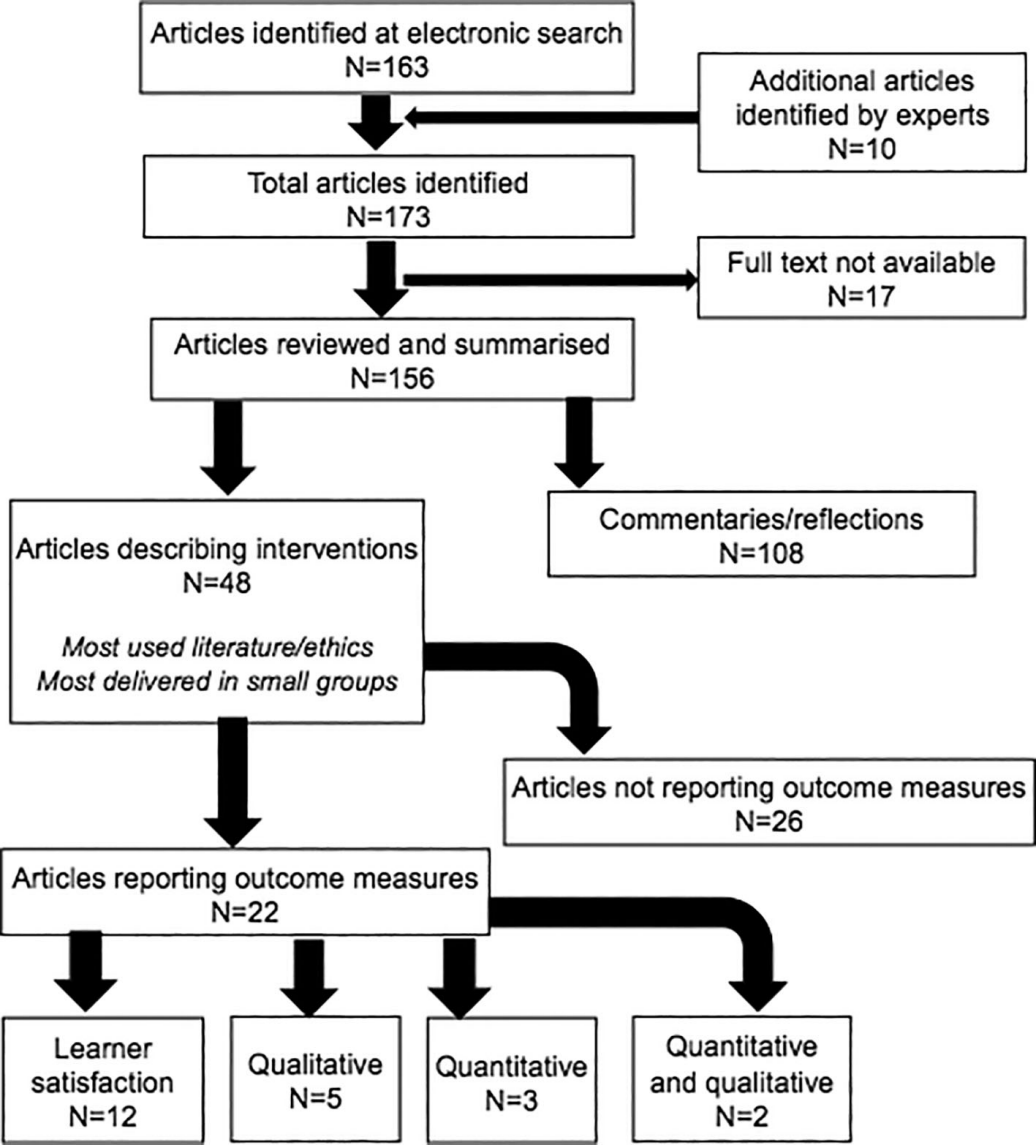
Flow diagram of search

The search yielded 163 titles. Discussion with experts in the field yielded an additional 10 articles for review. Of these 173 articles, 17 were excluded because a full text was not available, yielding a total of 156 articles. See Figure 1 for a summary of the results.

Of the 156 articles reviewed, most (108; 69.2%) were commentaries and reflections on the humanities as they relate to medicine. Forty-eight articles described a humanities-based intervention, most of which were implemented in high-income countries. Although this search did identify articles - at the abstract review level - from low/middle income countries on humanities in medicine, the full-text of these articles was not always available in the English language. Of those articles that were available in English, most took the form of a commentary or reflection rather than a report of an intervention. Twenty-six of the 48 intervention articles did not report any formalised evaluation outcomes ( Goodwin and Machin, 2016; Kemp and Day, 2014; Ortega, Andreoli and Chima, 2011; Wald *et al.*, 2010; Joachim, 2008; Kumagai, 2008; Boudreau, Cassell and Fuks, 2007; Meites, Bein and Shafer, 2003; Louis-Courvoisier, 2003; Frich and Fugelli, 2003; Acuna, 2003; Murray, 2003; Jones and Verghese, 2003; Hawkins, Ballard and Hufford, 2003; Wear, 2003; Fried, Madar and Donley, 2003; Spike, 2003; Krackov *et al.*, 2003; Sirridge and Welch, 2003; Andre *et al.*, 2003; Montgomery, Chamers and Reifler, 2003; Kirklin, 2003; Rizzolo, 2002; Sklar *et al.*, 2002; Downie *et al.*, 1997; Self and Baldwin, 1990). Of the 22 articles reporting evaluation outcomes, 12 described learner satisfaction outcomes (del Pozo and Fins, 2005; Wald *et al.*, 2015; Gurtoo *et al.*, 2013; Abdel-Halim and AlKattan, 2012; George and Dellasega, 2011; Karnad, 1999; Shapiro, 2003; Newell and Hanes, 2003; Anderson and Schiedermayer, 2003; Lypson and Hauser, 2002; Bertman and Marks, 1985; Wilson and Blackwell, 1980). Only ten articles included any formal method of evaluation or assessment of impact beyond learner satisfaction. Of those ten articles, five used qualitative techniques to evaluate learners (Thompson *et al.*, 20156; Ramani and Orlander, 2013; Gulpinar, Akman and User, 2009; Wachtler, Lundin and Troenin, 2006; Bonebakker, 2003), three used quantitative measures ( Rodriguez *et al.*, 2013; Wiecha and Markuns, 2008; Wiecha, Vanderschmidt and Schilling, 2002), and two used a combination ( Shapiro, Morrison and Boker, 2004; Shapiro *et al.*, 2005). Quantitative measures were typically Likert scales or validated empathy scales (Empathy Construct Rating Scale and the Balanced Emotional Empathy Scale) ( Shapiro, Morrison and Boker, 2004).

A number of areas within the humanities were utilized in curricula, but the most common type of intervention was one based around literature or ethics, with fewer interventions using the visual arts or performing arts. The most common form of teaching delivery was small group teaching, and interventions were often facilitated by a professional with humanities experience ( George and Dellasega, 2011; Wald *et al.*, 2010; Gulpinar, Akman and User, 2009; Boudreau, Cassell and Fuks, 2007; Wachtler, Lundin and Troenin, 2006; Karnad, 1999; Shapiro, Morrison and Boker, 2004; Hawkins, Ballard and Hufford, 2003; Andre *et al.*, 2003; Shapiro, 2003; Newell and Hanes, 2003; Anderson and Schiedermayer, 2003). Only three interventions utilized newer methods of medical education such as websites to curate content, or social media to enable more frequent communication between students ( Wiecha and Markuns, 2008; Wiecha, Vanderschmidt and Schilling, 2002, George and Dellasega, 2011).

Two of the educational interventions incorporated humanities teaching into an anatomy course to enable students to begin to understand the patient perspective of illness along with learning techniques of dissection ( Bertman and Marks, 1985; Rizzolo, 2002). Of note, few of the 48 articles described an intervention delivered in a clinical environment or in the presence of a patient, using related humanities material (such as examples from literature describing a patient’s experience of a certain illness) to help students comprehend the impact of different illnesses on patients ( Ramani and Orlander, 2013; Kumagai, 2008; Wilson and Blackwell, 1980; Gurtoo *et al.*, 2013). Curricula were predominantly delivered in either a didactic or seminar-based format with no patient involvement, and many were elective courses. One intervention used social media (including Twitter, YouTube and Skype) to augment classroom teaching ( George and Dellasega, 2011), while another hosted the content of a humanities clerkship on a website ( Wiecha and Markuns, 2008), enabling students to access materials at times of their choosing.

Certain challenges were frequently identified throughout the literature. Lack of funding was a commonly cited problem, resulting in humanities curricula that could not be guaranteed a long-term place in medical training. Another common problem was difficulty scheduling the teaching amongst the multitude of other academic commitments held by learners.

## Discussion

### Summary of findings

Assessing the landscape of the literature on the integration of humanities curricula into medical education revealed that the majority of articles were not reports of original research; rather they were opinion pieces discussing the relationship between humanities and medicine, or arguing for the inclusion of humanities teaching within medical education. Forty-eight of these articles described a curricular intervention, but only 22 included any outcomes measurements on trainee knowledge, attitudes or behaviours. A systematic approach to curriculum design was often lacking. None of the articles assessed the impact of humanities curricula on patients, or evaluated patient care outcomes.

### Strengths and Limitations

A strength of this review is the assistance of an informationist to identify the terms used to search the electronic data base. The consultation with expert colleagues to identify any articles missed by the search could be viewed as a strength or as a limitation, as any expert has the potential to introduce bias into a search. A clear limitation of this review is the decision to include only articles published in English, as we may have missed relevant articles on humanities curricula that were implemented in non-English speaking countries.

### Implications

Among the articles that described means of evaluating the effectiveness or impact of humanities interventions, qualitative feedback derived from learner interviews or written feedback was the most commonly used method. Although qualitative methodology enables participants to express themselves more freely and flexibly about issues and experiences that are important to them, it can be difficult to compare open-ended feedback from learners about educational interventions or to determine whether such interventions would be suitable for different population groups. The articles reporting use of a quantitative scale to evaluate impact typically used graded Likert ratings, although one study used the Empathy Construct Rating Scale (ECRS) and the Balanced Emotional Empathy Scale (BEES) ( Shapiro, Morrison and Boker, 2004). Although all of these scales use self-reported measures and therefore are subject to bias, they may be considered more objective than open-ended or written feedback, which is vulnerable to variability in interpretation among raters.

This review identified a number of significant gaps in the literature, the most important being a lack of outcome data. This significantly limits the evidence base for the use of humanities in medical training, and which may make it hard to argue for permanent embedding of humanities curricula and more widespread inclusions in medical education. Future studies should focus on evaluating the impact of humanities-based didactics on trainees, either in the form of qualitative data or using scales already described in the existing literature.

Additionally, many of the educational interventions were undertaken as elective courses, potentially creating a self-selected group of interested learners. This could skew any evaluation results towards a positive impact. Evaluation of humanities curricular interventions that are integrated into the general medical curriculum will be especially valuable in determining impact on the training of physicians. Since the goal of including humanities curricula is to help trainees become more humanistic clinicians, it is important that future studies pursue assessment of whether such a curriculum improves humanistic practice by doctors.

Most of the curricula were described as running separately to biomedical teaching on areas such as pathology, biochemistry or physiology. They were often taught by arts educators, without clinician involvement. This could limit the potential for students to understand how humanities can contribute to all areas of medicine as opposed to simply communication or writing skills. Integrated collaborative teaching, delivered by arts educators together with clinicians and involving an understanding of both the biomedical underpinnings of illness and the experience of illness itself, could help to bridge the gap between science education and humanities education. It could therefore help to illuminate its relevance for facilitating a more holistic understanding of patients.

Finally, although many of the interventions were delivered during medical training, and students therefore participated in separate humanities courses contemporaneously with meeting patients and involving themselves in the ward environment, very few of the interventions actually involved the patients themselves. Including patients in medical education has been shown to enhance the student learning experience ( Jha *et al.*, 2009; Ramani and Orlander, 2013). Speaking to patients, either in the classroom or at the bedside, could help students connect the ‘standard patient’ with classic symptoms described in medical textbooks to the patient read about in assigned literature or viewed in art. Effective humanities teaching also includes time for reflection and focused mentoring to ensure positive learning experiences are gained ( Stern *et al.*, 2008). The human connection engendered in humanities interventions may help preserve empathy felt towards patients, which has been identified previously as an area of importance ( Pedersen, 2010; Stratta, Riding and Baker, 2016).

Future humanities medical education interventions would benefit from grounding in principles of curriculum design and inclusion of formal evaluation of learner satisfaction, knowledge, and attitudes and behaviour towards patients. If possible, patients should be involved in curricular design and/or implementation, and be able to give feedback to learners.

## Conclusions

The role of the humanities within medical education has been extensively discussed in theory, but very little has been done to evaluate its use in practice. This review identified a number of significant gaps in the literature, the most important being a lack of rigorous evaluation of curricular interventions that include outcome measures. Future studies should focus on gaining qualitative and quantitative data regarding impact of curricular interventions on learners and/or patients.

## Take Home Messages

• There is a need to help medical students and junior doctors retain empathy as they progress through training
• Humanities curricula have been shown to enhance reflection
• There is a lack of outcome data demonstrating impact on learner behaviour or the patient experience
• Collaborative teaching between clinicians, arts educators and patients may be considered in order to bridge the gap between science and humanities
• Challenges to long-term integration of humanities curricula include lack of funding and difficulty scheduling
• Future work should include assessment of the outcome of humanities-based educational interventions on medical learner and patient outcomes

## Notes On Contributors

Anna Kathryn Taylor is an academic foundation doctor at East Lancashire Hospitals NHS Trust and a Pathfinder Fellow at the Royal College of Psychiatrists. She has five years’ experience in medical education and curriculum development, focusing on global health, diversity training, and a holistic approach to patient care.

Susan Lehmann is associate professor of Psychiatry and Behavioral Sciences and Psychiatry Clerkship Director at the Johns Hopkins University School of Medicine in Baltimore, Maryland.

Margaret Smith Chisolm is associate professor of and Vice Chair for Education in Psychiatry and Behavioral Sciences at the Johns Hopkins University School of Medicine in Baltimore, Maryland.
