# Supplementary material for: Integrating humanities curricula in medical education: a literature review
Source: MedEdPublish (2016). 2018 Aug 10;6:90. Originally published 2017 Jun 5. [Version 2] doi: 10.15694/mep.2017.000090.2 (PMC10885258; doi:10.15694/mep.2017.000090.2)
Supplement: Peer review history

# Reviewer report: Reviewer response for version 2

**DOI:** 10.21956/mep.20219.r31310

**Published:** 2018-12-21

**Authors:** Ken Masters

**Affiliations:** 1 Sultan Qaboos University

This review has been migrated. The reviewer awarded 3 stars out of 5 I am happy that the authors have taken the reviewers’ comments on the first version into account, and have been able to address several of the issues. An area of weakness that does not appear to be addressed is that the paper searched only Medline. Although the response from the authors indicates that this was addressed (“Include the limitations of only one data base”), the limitations given in the paper do not state this: “A clear limitation of this review is the decision to include only articles published in English, as we may have missed relevant articles on humanities curricula that were implemented in non-English speaking countries.” What has been ignored is that Humanities' Academic databases have not been consulted.In addition, the Results are still rather poorly reported, but the authors have removed the claim of a systematic review, so that mitigates it somewhat.On the strength of the revision, I do recognise the improvements in the paper, although it is a pity that the authors missed the opportunity to deliver a more enlightening paper. Still, it is worth reading by those involved in medical education.

Reviewer Expertise:

NA

No decision status is available

# Reviewer report: Reviewer response for version 2

**DOI:** 10.21956/mep.20219.r31312

**Published:** 2018-08-21

**Authors:** Trevor Gibbs

**Affiliations:** 1 AMEE

This review has been migrated. The reviewer awarded 4 stars out of 5 This is a difficult and complex area, but evaluation of our "humanities interventions" are very important. I belief that the paper is much clearer now and show how lax we are in our evaluations, even though we believe something is important.I am happy now to recommend this paper to all those involved in the Humanities teaching

Reviewer Expertise:

NA

No decision status is available

# Reviewer report: Reviewer response for version 2

**DOI:** 10.21956/mep.20219.r31311

**Published:** 2018-08-13

**Authors:** Sandra Kemp

**Affiliations:** 1 Curtin Medical School, Curtin University

This review has been migrated. The reviewer awarded 2 stars out of 5 My comments relate to this version of the article. I have not read the earlier version but noted the reviewers’ comments. Therefore, I will highlight some areas for additional comment. I should indicate that I have an interest as my article (Kemp & Day 2014) was one of the articles included in the review.I think the value of a literature review like this is to synthesise, rather than describe. That is, the reader needs to understand what is important from the work done, what the work highlights, or what is yet to be investigated. Strengthening that focus would be useful.I would recommend that personal views are appropriately contextualised. For example, it seemed that interventions involving quantitative evaluation data were very desirable to the authors. This seemed to devalue complex program evaluation, drawing on social sciences-based methodology, which may be required for integrated curricula. Coherence across the arguments in the paper, underpinned by conceptual frameworks used in humanities, would be useful.I would like to clarify some detail in the statement: "Twenty-six of the 48 intervention articles did not report any formalised evaluation outcomes (Goodwin and Machin, 2016; Kemp and Day, 2014 ...). The article I wrote with Day focussed on guidance and practical suggestions for medical educators about how technology-enhanced learning design can support the learning outcomes associated with medical humanities. It was not an intervention, so ensuring all articles reviewed are classified correctly will strengthen any conclusions drawn.I agree that this area is important for medical educators and robust literature reviews can help to inform readers.

Reviewer Expertise:

NA

No decision status is available

# Reviewer report: Reviewer response for version 2

**DOI:** 10.21956/mep.20219.r31309

**Published:** 2018-08-11

**Authors:** Nandalal Gunaratne

**Affiliations:** 1 Faculty of Medicine, Wayamba University of Sri Lanka

This review has been migrated. The reviewer awarded 3 stars out of 5 Thank you for your effort to improve on your previous article and useful critical comments. The references are also useful. This revised article shows the difficulties including arts and humanities into a curriculum in an effective manner.The other major issue is to identify it's impact. You have not made an attempt to identify a helpful way to do this. The questions that arise are1. Do we have two arms for comparison? One group randomly chosen to follow this curriculum and the other not doing so?2. How do we assess the impact on empathy and ethics as well as other end points?3. When do we do this? As students complete or during their internship/residency?Any views or personal experiences are welcome. Thank you.

Reviewer Expertise:

NA

No decision status is available

# Reviewer report: Reviewer response for version 1

**DOI:** 10.21956/mep.18980.r27191

**Published:** 2017-06-06

**Authors:** P Ravi Shankar

**Affiliations:** 1 American International Medical University

This review has been migrated. The reviewer awarded 2 stars out of 5 Thank you for the opportunity to review an interesting paper. As suggested by the other reviewers I do agree that this is more of a literature review of humanities programs in medical education. I have been active in humanities education in Nepal and the Caribbean for a decade. As a researcher and an educator from a developing country I do not see descriptions of humanities education from South Asia and other developing regions. The developing world is not well represented and I agree that though humanities programs are more common and better established in the developed world, these are becoming increasingly common in developing nations also. Measuring the impact of humanities curricula is a challenge in the developing world also though studies about the short term evaluation of the programs have been published. Educators in developing nations struggle with a number of challenges and hurdles while trying to incorporate humanities in medical education. Searching other databases in addition to PubMed will yield a greater selection of articles. The authors can provide more details about the search criteria used to identify papers selected for this review. The challenges and limitations suggested by the authors are relevant. I and my colleagues, predominantly from a medical background have facilitated humanities modules in Nepal. In Aruba in the Dutch Caribbean we had also had facilitators from a humanities and a psychology background. A standardized patient was also a co facilitator in the module. We were not able to directly bring the ‘patient voice’ into the sessions. I do agree with the authors that outcome data is weak and can be strengthened. We expect learning of the humanities to contribute to more empathetic and humane doctors. Obtaining long-term outcomes data is a challenge as there are a number of factors which can influence the behavior of doctors and humanities education is only one among the many influences.

Reviewer Expertise:

NA

No decision status is available

# Reviewer report: Reviewer response for version 1

**DOI:** 10.21956/mep.18980.r27194

**Published:** 2017-06-05

**Authors:** Subha Ramani

**Affiliations:** 1 Harvard Medical School, Brigham and Women's Hospital

This review has been migrated. The reviewer awarded 3 stars out of 5 I agree with my fellow reviewers that the subject is timely and important to address, but I was left with the question, "what are the take-home messages for me". The authors should be commended for tackling this topic and it is very clear that they have reviewed the literature in great depth. " Needs assessment" in the title is misleading, should be more like- what are the gaps in this field?The abstract is very well written.Details of the search are not available under methods, perhaps figure 1 was meant to help with this- alas it is missing.The results section communicates fairly well the existing types of articles on humanistic curricula. Reading between the lines, I gathered that most of them were commentaries, a systematic approach to curriculum design was often lacking and program evaluation was either completely missing or touched upon Level 1 of Kirkpatrick- satisfaction. However, we should not have to read between the lines and these points could be emphasized more.The discussion mirrored the results in many parts and I would have liked to have seen- what next and why it matters.I agree with the take home messages overallWith the time that the authors have invested in this project, I highly recommend that they think of writing a 12 tips paper which would be much more useful for all educators interested in this subject or planning to design humanistic curricula.

Reviewer Expertise:

NA

No decision status is available

# Reviewer report: Reviewer response for version 1

**DOI:** 10.21956/mep.18980.r27192

**Published:** 2017-06-05

**Authors:** Trevor Gibbs

**Affiliations:** 1 AMEE

This review has been migrated. The reviewer awarded 2 stars out of 5 A rather complicated paper that attempts to bring to the forefront of relevance a very important subject. I found it rather complicated to read because of the way that the authors put together their paper which did not flow and seemed to come to conclusions, within their Take Home messages section, not supported by findings in their text.Like my co-reviewer I wondered about how it was a needs assessment ( so would think that the title is not appropriate) and how much of an in-depth systematic review this was. To have experts providing another 10 significant papers without wondering where these papers came from ( maybe non-medical literature) might have given a new viewpoint on the search characteristics and might have eventually provided more papers. I would not wish to re-iterate my co-reviewers comments other than to add that I was left wanting at the end of this paper- I was really looking for the"so what" factor- the transference of this large amount of hard work into a tangible way forward

Reviewer Expertise:

NA

No decision status is available

# Reviewer report: Reviewer response for version 1

**DOI:** 10.21956/mep.18980.r27193

**Published:** 2017-06-05

**Authors:** Ken Masters

**Affiliations:** 1 Sultan Qaboos University

This review has been migrated. The reviewer awarded 2 stars out of 5 Although this paper has approached a very interesting and timely topic, the authors have made fundamental errors that severely reduce the value of the paper.TitleI’m not sure that the paper really does describe a Needs Assessment. It is a Literature Review, and it would be better if the title reflected that.Methodology• As this is a systematic literature review (“systematic search”), I would like to see far more details of the search process, which is standard in such a search, beginning with the description of a search protocol and then the search processes (e.g. number of articles/abstracts on initial search, exclusions and exclusion criteria, etc.), and accompanied by a flow-chart showing the process. Without that, it is just a search, and there is nothing to tell us that it was indeed systematic. (The paper does refer to a Figure 1, but no Figure 1 has been supplied, so this comment may or may not be valid).• Confining the search to PubMed/Medline only is extremely limited (although possibly off-set to some extent by the consultation with colleagues). The very nature of Humanities in medical education calls for a search of Humanities’ databases. Searching for Humanities articles without using Humanities databases is strange indeed (the equivalent of searching for medical articles without using Medline), and undermines the validity of the search. When performing an interdisciplinary study of any type, it is crucial to bear all disciplines in mind. Results:The Results reporting could have benefitted by having the results displayed in tables, along the lines of a meta-analysis. One of the strengths of a literature review is that the reader can see the value of each paper at a glance: the way that these results have been reported makes it exceedingly difficult to do so. The authors are also sometimes very loose in their statements that require a more detailed explanation, leaving the reader confused or with no real information: • “the most common type of intervention was one based around poetry, prose or ethics.” Poetry and prose are forms of writing, while ethics is a topic or subject. Placing these three into a sentence like this without further explanation of how these are connected or contrasted makes little sense. There is also little sense of what was actually done with these interventions or how they were used or addressed. For example, instead of simply referring to a poem, if the students had been taught poetics or poetry writing, then that would alter its impact, but, the way it is currently described leaves the reader with no real insight into what was done. • “Two of the educational interventions incorporated humanities teaching into an anatomy course to enable students to begin to understand the patient perspective of illness along with learning techniques of dissection.” Again, while this is a good start, the reader is left hanging with little sense of what was actually done, apart from the incorporation of “humanities teaching” into the course, which is extremely vague.• “Of note, few of the 48 articles…” “Few” is not a number to be reported in the results. The same is to be said for “predominantly” in the next sentence. Accurate numbers should be given; these words can be used in the discussion, if you wish, but only after the actual numbers have been given in the Results.Discussion:The first part of the Discussion introduces new results, again, though, with vague terminology (the majority, few, very small minority). The second part of the Discussion shows greater potential for insight, but is undermined by the poor Results reporting.So, while the paper has tackled a worthwhile topic, the Title is misleading, there is a flaw in the study design (not examining any Humanities’ databases), poor results reporting (the effect of which may or may not be reduced by the inclusion of the missing Figure 1), and a Discussion that is part Result-reporting and part Discussion. The authors have made a worthwhile effort, but the paper really does need quite a bit of work.

Reviewer Expertise:

NA

No decision status is available
